# GreenLeafVI: A FIJI Plugin for High‐Throughput Analysis of Leaf Chlorophyll Content

**DOI:** 10.1111/ppl.70588

**Published:** 2025-10-19

**Authors:** Thalia Luden, Jelmer van Lieshout, Sarah L. Mehrem, Basten L. Snoek, Joost Willemse, Remko Offringa

**Affiliations:** ^1^ Plant Developmental Genetics Institute of Biology Leiden, Leiden University Leiden the Netherlands; ^2^ Theoretical Biology and Bioinformatics, Biodynamics and Biocomplexity Utrecht University Utrecht the Netherlands; ^3^ Cell Observatory Institute of Biology Leiden, Leiden University Leiden the Netherlands

**Keywords:** chlorophyll content, high‐throughput phenotyping, image‐based quantification, ImageJ, leaf senescence

## Abstract

Chlorophyll breakdown is a central process during plant senescence or stress responses, and leaf chlorophyll content is therefore a strong predictor of plant health. Chlorophyll quantification can be done in several ways, most of which are time‐consuming or require specialized equipment. A simple alternative to these methods is the use of image‐based chlorophyll estimation, which uses the color values in RGB images to calculate colorimetric visual indexes as a measure of the leaf chlorophyll content. Image‐based chlorophyll measurement is non‐destructive and requires no specialized equipment, apart from a digital camera. Here, we developed the ImageJ plugin Green Leaf Visual Index that facilitates high‐throughput image analysis for quantifying leaf chlorophyll content. Our plugin offers the option to white‐balance images to decrease variation between images and has an optional background removal step. We show that this method can reliably quantify leaf chlorophyll content in a variety of plant species. In addition, we show that image‐based chlorophyll quantification can replicate Genome‐Wide Association Study results based on traditional chlorophyll extraction methods, showing that this method is highly accurate.

## 1 Introduction

Plant health can, in many cases, be deduced from the leaf chlorophyll (Chl) content, as several stress factors can affect the photosynthetic capacity of plants. The Chl content can be reduced in response to stresses, such as drought, high salt, nutrient deficiency, or pathogen infection, or as a result of programmed plant senescence (Hörtensteiner and Kräutler 2011). Quantification of Chl is therefore an important tool to monitor plant health in a variety of conditions (Kalaji et al. 2017; Wang et al. 2022). Various methods for measuring Chl content are available, such as solvent‐based Chl isolation like acetone extraction followed by photospectrometry at 645 and 663 nm (Arnon 1949), Soil Plant Analysis Development (SPAD) measurement (Yadava 1986; Markwell et al. 1995; Konica Minolta Optics I 2009), Chl fluorescence measurement (Murchie and Lawson 2013; Legendre et al. 2021), hyperspectral imaging (Zhang, Ge, et al. 2022; Taha et al. 2024) and image‐based colorimetric visual index (CVI) estimations of Chl (Ali et al. 2012; Bresson et al. 2018; Guo et al. 2020; Guendouz et al. 2021; Taha et al. 2024). The acetone‐based Chl extraction method directly measures Chl content (mg Chl cm^−2^ or in mg Chl mL^−1^), and is one of the most accurate ways to quantify Chl. However, this method is labor‐intensive and destructive and requires specialized equipment to measure the OD645 and OD663. Due to the destructive nature of this method, it is also poorly suited to monitor Chl content over a plant's lifetime or under changing conditions. Chl measurements with a SPAD meter are non‐destructive, but SPAD measurements remain labor‐intensive, as each measurement needs to be recorded manually, and SPAD values are strongly affected by the position on the leaf where the measurements are made. In addition, the SPAD meter can only be used on leaves above a certain size, which excludes the possibility of measuring young or small leaves, or plants grown in tissue culture that need to remain in sterile conditions. Chl fluorescence measurements and hyperspectral imaging methods are non‐destructive and can yield useful information, but require specialized equipment that can be expensive and are therefore not universally available to researchers. In addition, measuring Chl fluorescence makes use of the photo‐saturation of Chl followed by a cool‐down period, which makes this type of measurement relatively time‐consuming. In contrast, image‐based Chl quantification methods require only the use of a digital camera and uniform lighting, are non‐destructive, and are highly adaptable to different environments and experimental setups. Because no specialized equipment is required, image‐based Chl quantification can readily be applied in any environment and is almost universally available to all researchers. Moreover, digital images can be easily processed in batch, allowing high‐throughput monitoring of Chl content in a variety of experimental setups (Prakash Yadav et al. 2010; Zhang et al. 2018; Zhang, Ge, et al. 2022; Fernando Sánchez‐Sastre et al. 2020; Guo et al. 2020; Özreçberoğlu and Kahramanoğlu 2020; Guendouz et al. 2021; Taha et al. 2024).

Image‐based Chl quantification relies on the relative pixel intensity in the Red, Green, and Blue channels of RGB images, from which Chl content can be deduced by using different mathematical models (Woebbecke et al. 1995; Kawashima and Nakatani 1998; Guo et al. 2020; Taha et al. 2024). Alternatively, values in the hue, saturation value color‐space (HSV) can be measured (Sass et al. 2012; Bresson et al. 2018). Various studies have shown the usefulness of RGB image analysis and their derived CVIs in estimating Chl content in different species, and strong correlations with total Chl content have been shown for many plant species. For example, Kawashima and Nakatani (1998) showed that the Chl content of wheat and rye leaves could be measured by using a video camera and showed that the best method to quantify Chl content was the normalized difference between the Red and Blue values (R − B)/(R + B), henceforth referred to as the Kawashima index. Other well‐performing models that were tested by Kawashima and Nakatani include the normalized Red, Green, and Blue indexes (R/[R + G + B], G/[R + G + B], B/[R + G + B]), and the difference between red and blue divided by the sum of all channels ([R − B]/[R + G + B]). Ali et al. (2012) showed that the Kawashima index correlated strongly with SPAD‐502 readings in tomato, and Taha et al. (2024) showed the same in hydroponically grown lettuce. Similarly, Fernando Sánchez‐Sastre et al. (2020) showed that the highest‐scoring models tested by Kawashima and Nakatani (1998) also showed a strong correlation with the Chl content (measured using Chl fluorescence) in sugar beet leaves. On the other hand, Ibrahim et al. (2021) and Ali et al. (2012) found that the Green:Red ratio (G:R) correlated with SPAD‐502 Chl measurements in lettuce more strongly than the Kawashima index. Other researchers have used custom models to link the RGB color values to Chl content. For example, Prakash Yadav et al. (2010) showed that Chl content quantified by SPAD‐502 could be measured in micro‐propagated potato with similar accuracy when using RGB images as well as by the hue, saturation, and intensity values derived from these images, and a similar approach was used in Sorghum by Zhang, Ge, et al. (2022). Other applications of the RGB color space have also been applied, for example, by Govindasamy et al. (2017), who used RGB images to monitor the symbiosis efficiency between rhizobia and soybean plants, and Bu et al. (2024), who used RGB parameters to measure soybean pod freshness. Han et al. (2021) used aerial images to monitor the growth of *Hibiscus cannabinus* based on RGB‐derived parameters, and Zhang et al. (2018) used a similar approach for maize. Finally, Barraza‐Moraga et al. (2022) applied RGB analysis to satellite images to measure the Chl‐A content of algae in Lake Lanalhue in Chile. Overall, these examples show that RGB image analysis can successfully be applied in plant research and is a feasible method of Chl measurement.

Digital phenotyping relies on careful image acquisition and processing. The open‐source software FIJI is a distribution of the image analysis program ImageJ, which is a commonly used and highly customizable tool for image analysis in the life sciences (Schindelin et al. 2012). FIJI offers the possibility of automating analysis steps via macros or custom‐made plugins. In recent years, several tools have been developed to quantify leaf Chl content from digital images, such as the Python‐based program plantCV (Gehan et al. 2017; Casto et al. 2022) or ImageJ plugins such as the one developed by Liang et al. (2017). In other cases, researchers made use of custom‐made analysis tools, such as in MatLab (Ali et al. 2012; Perez‐Patricio et al. 2018; Taha et al. 2024). Despite their usefulness, these tools present certain drawbacks for researchers wishing to use RGB images for Chl quantification. PlantCV, while highly customizable and suitable for high‐throughput analyses, is based on the Python language and requires a degree of familiarity with this programming language before it can be applied. On the other hand, many biologists are already familiar with the user interface of FIJI/ImageJ, which can be used without prior knowledge of programming languages due to its graphical user interface (Schindelin et al. 2012; Schneider et al. 2012). However, the existing tools available for leaf image analysis in ImageJ either do not quantify leaf Chl content (e.g., LeafJ by Maloof et al. 2013), or require several manual image calibration steps (Liang et al. 2017), making them unsuitable for high‐throughput analysis of Chl. To our knowledge, there is no tool allowing for high‐throughput analysis of RGB images for direct quantification of Chl content that does not require programming skills. We therefore set out to develop a FIJI plugin that reliably quantifies leaf Chl content based on digital images in a high‐throughput manner for various applications, which we named Green Leaf Visual Index (GreenLeafVI). GreenLeafVI offers the option to normalize image brightness, segment images to reduce background noise, and calculate the RGB values of multiple objects per image, along with various methods of leaf Chl content quantification, and can process images in batch mode, making it suitable for high‐throughput image processing. The output data is stored in a tidyR‐compatible format that can readily be used for further statistical analysis in R or other statistical software. We show that GreenLeafVI can be applied to quantify Chl content in different plant species and can accurately reproduce Genome‐Wide Association Study (GWAS) results obtained by traditional Chl quantification methods in lettuce, validating its use for high‐throughput phenotyping experiments.

## 2 Material and Methods

### 2.1 Plant Materials and Growth Conditions

*Arabidopsis thaliana* (Arabidopsis) ecotype Col‐0 seeds were sown on humid soil (90% turf, 10% sand) and stratified at 4°C for 3 days and subsequently transferred to a growth chamber with long‐day conditions (16/8 h light/dark) at 21°C and 65% relative humidity. Seedlings were repotted to individual pots after 7 days, and the fifth leaf of each plant was harvested 23 days after the end of stratification. Leaves incubated for 0–5 days in the dark were used for senescence measurements to represent various stages of leaf senescence.

For testing the correlation between colorimetric measurements and Chl content, *Lactuca sativa* (lettuce) cv. Cobham Green seeds were sterilized in 50% chlorix bleach and 50% MilliQ solution and stratified in distilled water for 4 days at 4°C. Subsequently, seeds were sown on moist soil (90% turf, 10% sand) and transferred to a growth chamber with long‐day conditions (16/8 h light/dark) at 21°C and 70% relative humidity. The fourth leaf was harvested 10 days after emergence, and 25 mm Ø leaf disks were taken and incubated in demineralized water with 1% agarose in the dark for up to 5 days. Leaves subjected to different dark incubation periods were used for Chl quantification to represent a range of senescence stages. For the GWAS experiment, we selected a total of 184 *Lactuca sativa* accessions as described by Dijkhuizen et al. (2025). Seeds were treated as described above, and leaf disks of the fourth leaf from three plants per cultivar were taken at 10 days after leaf emergence and used directly for imaging and Chl extraction.

*Nicotiana benthamiana* (tobacco) seeds were sown on moist soil (90% turf, 10% sand) and germinated in a growth chamber with long‐day conditions (16/8 h light/dark) at 21°C and 70% relative humidity. After 7 days, seedlings were repotted into individual pots. After 3 weeks, mature leaves were detached and incubated in large petri dishes on demineralized water with 0.5% agarose in the dark for up to 7 days.

*Solanum lycopersicum* (tomato) cv. Moneymaker seeds were sterilized in bleach and germinated on solid MS medium and transferred to soil after 3 weeks. Plants were grown in long‐day conditions (16/8 h light/dark) at a 24/18°C day/night temperature regime. Leaves were harvested from flowering plants and incubated in large petri dishes on demineralized water with 0.5% agarose in the dark for up to 7 days, with harvesting points between 0 and 7 days of dark incubation.

### 2.2 Senescence Induction, Imaging, and Chl Isolation

Arabidopsis leaves were floated on 5 mM MES buffer (pH 5.6) in 5 cm Petri dishes sealed with parafilm, wrapped in aluminum foil, and kept at 21°C for up to 5 days. Lettuce, tomato, and tobacco leaves were harvested at various ages and placed in Petri dishes with demineralized water with 0.5% agarose, sealed with parafilm, wrapped in aluminum foil, and stored at 21°C for up to 7 days. Leaves were imaged at different time points between 0 and 7 days of dark incubation, and leaf disks were collected for Chl extraction after imaging. All images were taken with a Nikon DC3000 DSLR camera under uniform white light. Plant leaves were placed on a homogeneous white or black background along with a white square that served as a reference for image brightness.

Chl extraction was performed according to the protocol of Arnon (1949). Briefly, 5 mm diameter round leaf disks were taken after imaging (2 per Arabidopsis leaf and 3 per leaf for other species) and placed in a 96‐well deep‐well plate along with a metal bead and stored at −80°C until extraction. For extraction, the leaf tissue was pulverized and resuspended in 200 μL 25 mM sodium phosphate buffer (pH 7) and 800 μL 80% (v/v) acetone. Samples were then incubated at room temperature in the dark for 1 h with gentle shaking and centrifuged for 10 min at 3000 *g*. Two hundred microliter of the supernatant was then transferred to a 96‐well transparent‐bottom plate, and the absorption (D) at 645 and 663 nm was measured with a Spark 10 M microplate reader (TECAN Group AG). The total Chl content in mg L^−1^ was then calculated with the following formula: Chl_Total_ = 20.2·D_645_ + 8.02·D_663_ as described by Arnon (1949). We decided to measure the Chl content in mg cm^−2^ rather than mg g^−1^ fresh weight to minimize variation between measurements because we observed that leaves often wilted after several days of dark incubation, reducing the fresh weight, whereas leaf area was less affected by the senescence process. To calculate the Chl content in mg cm^−2^, the mg Chl in 1 mL (the extraction volume for each sample) was divided by the total area of leaf tissue used for extraction.

### 2.3 Image Analysis

**FIGURE 1:**
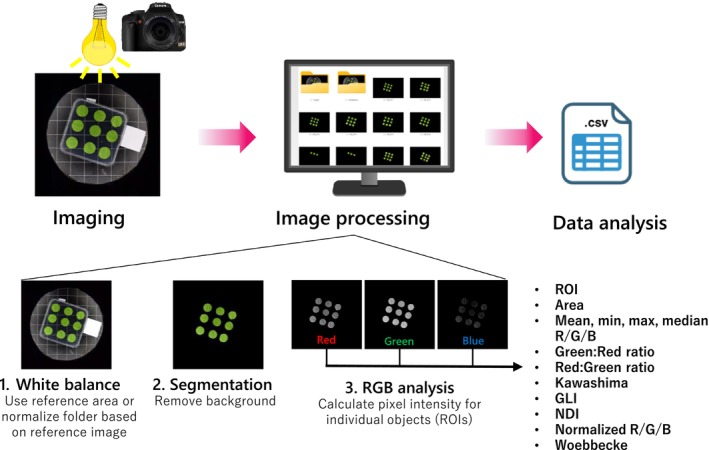
Workflow for high‐throughput Chl measurement with GreenLeafVI. Images are taken under homogenous light with a white reference area. The images are then batch‐processed for (1) white‐balancing based on the white reference area; (2) segmentation to remove the background; and (3) measurement of pixel intensity in the Red, Green, and Blue channels. The results of the RGB analysis are stored in a results.csv file, which, in addition to the RGB values, also contains the region of interest (ROI), the area of the objects, and several colorimetric visual indices (CVIs).

All images were analyzed using the FIJI open‐source release of ImageJ2 (Schindelin et al. 2012), with the custom GreenLeafVI plugin that can perform white balancing, automatic selection of leaves and removal of background, and RGB pixel intensity measurements semi‐automatically. The plugin is described in detail in Protocol S1, and Figure 1 shows a schematic overview of the steps performed by the plugin.

#### 2.3.1 White Balancing

In order to calibrate the image brightness and reduce inter‐image variation, we normalized the pixel intensity to the white reference area included in each image (Figure 1). This was done by splitting the image into Red, Green, and Blue channels and automatically measuring the mean pixel intensity in the white reference area. Next, the pixel adjustment factor for each of the channels was calculated by dividing the maximum brightness (255 for 8‐bit images) by the mean pixel intensity of the white area (adjustment factor = 255/mean). The white reference was then set to an intensity of 255 in all three channels, and the pixels outside of the reference area were normalized based on the adjustment factor for each channel and re‐stacked into an RGB image with a “_whitebalanced” suffix for further analysis.

#### 2.3.2 Segmentation of Images to Reduce Background

In order to exclude measurements of non‐leaf objects or areas in the background of the image, we included an optional segmentation step in the GreenLeafVI plugin (Figure 1). The segmentation step extracts leaf‐like objects from the background by creating a mask covering the leaves based on minimum and maximum HSV and minimum area parameters, and removes the background by setting the pixel intensity outside of masked areas to 0. The HSV values used to distinguish leaves from the background were calibrated manually for each species. The images were then saved, extended by a “_segmented” suffix for further analysis. We chose HSV as a color model for segmentation as it not only filters for color (Hue) but also incorporates intensity (Saturation) and lightness (Value), improving object‐to‐background segmentation.

#### 2.3.3 RGB Analysis and Color‐Based Methods of Chl Estimation

To measure the R, G, and B pixel intensities of individual objects in an image, a mask selecting individual objects was made, as in the segmentation step. The original image was then split into Red, Green, and Blue channels, and the minimum, maximum, median, and mean pixel intensity was measured in each object of the mask covering the leaves (Figure 1). The mean values of each leaf measured in the Red, Green, and Blue channels were used to calculate the different visual indexes: Green:Red ratio (GR_ratio), Red:Green ratio (RG_ratio), Kawashima index (Kawashima), Green Leaf Index (GLI), Normalized Difference Index (NDI), normalized Red (Red_norm), normalized Green (Green_norm), normalized Blue (Blue_norm), and the Woebbecke index (Woebbecke).

### 2.4 Data Analysis

#### 2.4.1 Correlation Analyses

Data generated by the ImageJ plugin and from spectrophotometry results were analyzed in R (R Core Team 2023). Pearson's correlation analysis between the different colorimetric indexes and the total Chl content as mg Chl cm^−2^ was performed with the R package psych (Revelle 2023), and plots were generated by using the ggplot2 (Wickham 2016) and ggpubr packages (Kassambara 2023).

#### 2.4.2 GWAS Analysis

SNP data were obtained from Dijkhuizen et al. (2025). Kinship was computed as the covariance matrix of SNPs using the cov() function in R. SNPs were filtered for a MAF > 0.05. For GWAS, we used R version 4.2.2 and the lme4QTL package (Ziyatdinov et al. 2018). GWAS was performed on the filtered SNP set using a linear mixed model (relmatLmer and matlm), with the kinship matrix included as a random effect. The Bonferroni method was used to correct for multiple testing, with the Bonferroni‐corrected significance threshold being −log10(0.05/2,485,803) > 7.69. Manhattan plots were created using ggplot (Wickham 2016). Quantile–Quantile (Q–Q) plots were created using the ggfastman R‐package (Tremmel 2021). The genome annotation and gene function prediction of the Salinas reference genome V8 were used for annotation of significantly associated loci (Reyes‐Chin‐Wo et al. 2017).

## 3 Results and Discussion

### 3.1 Normalized Red and Green:Red Ratios Are Accurate Predictors of the Chl Content in Leaves

**FIGURE 2:**
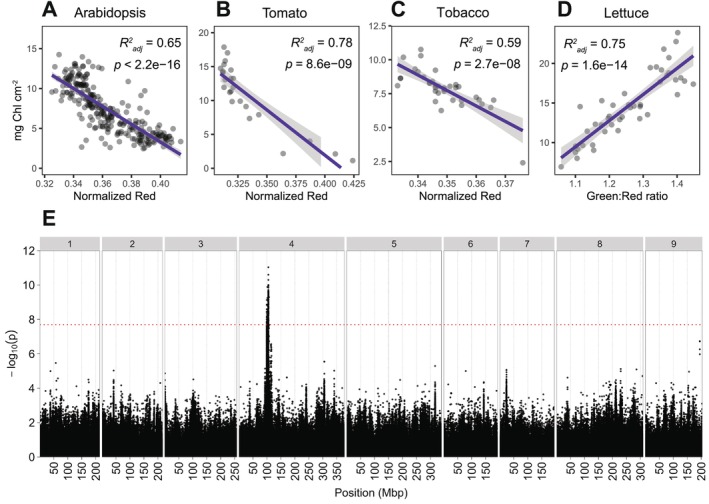
Application of CVIs for measuring Chl content in different species. (A–D) Correlations between total Chl content as mg Chl cm^−2^ and the best‐scoring CVI for each species. (A) Arabidopsis, (B) tomato, and (C) tobacco each show the strongest correlation between mg Ch·cm^−2^ and the normalized Red value (Rn). (D) In lettuce, mg Chl·cm^−2^ correlates best with the Green: Red ratio (G:R). (E) Manhattan plot of the GWAS on the Green: Red ratio measured on the fourth leaf of 184 lettuce cultivars. Genomic position, indicated in megabasepairs (Mbp), is shown on the *x*‐axis, chromosome numbers are indicated on top. Significance as –log_10_(*p*) is shown on the *y*‐axis. The Bonferroni threshold of −log_10_(*p*) > 7.69 is indicated by the red horizontal line.

We designed the GreenLeafVI plugin as a tool to easily phenotype the Chl content in different plant species. We therefore ran the three steps of the GreenLeafVI plugin (white balancing, segmentation, and RGB analysis) on four different plant species: Arabidopsis, tobacco, tomato, and lettuce. In addition, we measured the Chl content (in mg cm^−2^) in extracts from each imaged leaf to test how well the different CVIs calculated by the GreenLeafVI plugin correlated with the Chl content in mg cm^−2^. To assess which CVI most accurately quantifies the Chl content, we performed linear regression analysis with several previously described CVIs and the Chl content in mg cm^−2^ as determined by the classical extraction method for the four plant species. Our data show that several CVIs show a strong correlation with the content of total Chl (Chl A and Chl B, in mg cm^−2^) for these different plant species (Figure 2A–D). Overall, the CVI‐based quantification of Chl was most accurate for lettuce, where the Green:Red ratio (G:R) and the Normalized Difference Index (NDI; [Rn − Gn]/[Rn + Gn + 0.01]) were the most robust proxies of Chl content, followed by the normalized Red value (Rn; R/[R + G + B]) (Figure 2D, Table 1). For Arabidopsis, tobacco, and tomato, the normalized Red value best correlated with the Chl content, although we also found a strong correlation with the NDI and G:R (Figure 2A–C, Table 1). Interestingly, we observed that the correlation of CVIs and Chl content in mg cm^−2^ differed between species, with lettuce showing the best overall correlation, and tobacco the weakest, suggesting that CVIs are more suitable for quantifying Chl levels in some species compared to others. Previous research has shown that G:R is a good proxy of Chl content in lettuce (Taha et al. 2024) and that Rn shows the best consistency between species (Ali et al. 2012). Taken together, our data show that overall, Rn, G:R, and NDI are the best proxies of Chl content and can be reliably used to compare Chl levels in different samples.

**TABLE 1:** Correlations of various CVIs with the chlorophyll content in mg Chl·cm^−2^ (linear regression).

Index	Formula	Arabidopsis		Tomato		Tobacco		Lettuce	
		*R* ^2^	*p*	*R* ^2^	*p*	*R* ^2^	*p*	*R* ^2^	*p*
Green:Red ratio	G/R	0.607	< 2.2e−16	0.739	4.55E−08	0.3627	6.10E−05	**0.752**	**1.61E−14**
Kawashima Index	(R − B)/(R + B)	0.205	1.33E−13	0.551	2.00E−05	−0.027	0.7600	0.195	0.0016
Normalized Red (Rn)	R/(R + G + B)	**0.647**	**< 2.2e−16**	**0.775**	**8.62E−09**	**0.590**	**2.72E−08**	0.673	5.92E−12
Normalized Green (Gn)	G/(R + G + B)	0.343	< 2.2e−16	0.481	1.04E−04	0.204	0.0034	0.646	3.11E−11
Normalized Blue (Bn)	B/(R + G + B)	0.009	0.0755	0.092	0.0821	0.005	0.2876	0.363	9.02E−06
Normalized Difference Index (NDI)	(Rn − Gn)/(Rn + Gn + 0.01)	0.608	< 2.2e−16	0.739	4.45E−08	0.370	4.98E−05	**0.754**	**1.37E−14**
Green Leaf Index (GLI)	(2G − R – B)/(2G + R + B)	0.339	< 2.2e−16	0.487	9.10E−05	0.203	0.0034	0.649	2.60E−11
Woebbecke Index*	(G − B)/(R − G)	0.638	< 2.2e−16	0.822	6.603E−10	0.513	5.49E−07	**0.768**	**4.03E−15**

*Note:* In the formula column, R, G, and B stand for average Red, Green, and Blue pixel intensity values. Curves were fitted with a linear model fitting y~x, with *y* representing mg Chl cm^−2^ and *x* the CVIs in all cases except for the Woebbecke Index (*), where the curve was fit to a model of $y\sim -\frac{1}{x}$. For each plant species the CVIs correlating best with the Chl content are highlighted in bold.

Surprisingly, the correlation between the Chl content and the Kawashima index was not as strong as shown in some earlier reports (e.g., Kawashima and Nakatani 1998; Taha et al. 2024), and was outperformed by G:R, NDI, and Rn in all four species. Similar results were obtained by Guo et al. (2020), who showed that both the Kawashima and Woebbecke Indexes were outperformed by G:R and GLI when using aerial RGB images to quantify the Chl content in field‐grown maize. Ali et al. (2012) showed that the Kawashima index performed well in tomato, but not in lettuce or broccoli, whereas Rn performed well and was most consistent between species, a finding that is reinforced by our results. One possible explanation for the poor performance of the Kawashima index compared to Rn and other indexes could be the omission of G in the Kawashima index formula ([R − B]/[R + B]; Table 1), despite the green color of Chl. This corresponds with the fact that each of the best‐performing indexes in our setup (Rn, G:R, NDI) included green pixel intensity in their formulas (Table 1), showing that the green value is of importance for Chl quantification. However, greenness alone was not sufficient to accurately quantify the Chl content, as the normalized Green value (Gn; G/[R + G + B]) showed only a moderate correlation with the actual Chl content in our experiments (Table 1) as well as in previous studies (Ali et al. 2012). Together with our finding that Rn and G:R are the top performing CVIs, the intensity in the green and especially red channels best reflects the Chl content, whereas the intensity in the blue channel provides additional, but not indispensable, information.

Upon initial linear regression analysis, the Woebbecke Index appeared to correlate very poorly with the Chl content. While most CVIs showed a linear correlation with the actual Chl content of the type *y* = *x* (with *x* = CVI, *y* = mg Chl cm^−2^; Figures S1–S7), the Woebbecke Index appeared to form a non‐linear, hyperbolic relation with the Chl content (Figure S8). A linear model using a regression curve fitting with a formula of the type *y* = −1/*x* (with *x* = Woebbecke Index, *y* = mg Chl cm^−2^) showed that the Woebbecke Index actually correlated more strongly than any other CVI with the Chl content in tomato and lettuce, with an *R* ^2^ of 0.801 in tomato and an *R* ^2^ of 0.768 in lettuce (Figure S2, Table 1). However, despite the high *R* ^2^ values implying the Woebbecke Index as the superior CVI, we observed that the Woebbecke Index sometimes caused extremely positive or negative values that were not reflected by the other CVIs or the Chl content in mg cm^−2^, which could complicate comparisons between different measurements. While for lettuce and tobacco the Woebbecke Index generated exclusively negative values in a relatively narrow range, we observed that the Woebbecke Index formula generated extremely low (< −200) or extremely high (> 400) measurements in senescent leaves of Arabidopsis and tomato (Figure S8). The Woebbecke Index formula is defined as the difference between the G and B channels divided by the difference between R and G ([G − B]/[R − G]; Table 1). Because of this, the Woebbecke Index can create two types of abnormal values: extreme outliers and unexpected positive values. In cases where the R and G values are similar, such as in senescent leaves, the denominator of this formula approaches 0, resulting in extreme outlier values that may be either positive (when R > G ≥ B or B > G ≥ R) or negative (when B > G and R ≥ G or G > B and G ≥ R). In addition, more mild but still unexpected positive values can be generated in situations where R > G > B or B > G > R. These unexpected positive values or extreme values complicate the comparison between groups, as the mean value of the measurements in one group will be strongly affected by such values. Therefore, while the Woebbecke Index shows a strong hyperbolic correlation with mg Chl cm^−2^ and might be suitable to compare greenness among healthy, non‐senescent plants where R and G values are sufficiently different, our results show that it is unsuitable for quantifying Chl in senescent, yellowing leaves. We therefore advise using more robust indexes such as Rn, NDI, or G:R.

For this study, we used leaves at various stages of senescence (non‐senescent up to completely senesced) for Chl measurements and imaging. By including leaves in a range of senescence stages, we generated a dataset that is representative of a variety of conditions, where yellowing can represent different types of stress. Previous research (Woebbecke et al. 1995; Liang et al. 2017) largely focused on measuring the Chl content in healthy and/or young plants to monitor plant health during early development. Although this has yielded well‐performing CVIs, the omission of senescent or otherwise yellow leaves in the index calibration could explain why some indexes (e.g., Kawashima or Woebbecke) perform less in our conditions compared to previous studies. In addition, most studies have made use of SPAD‐502 readings to calibrate or measure the success of a CVI (e.g., Ali et al. 2012; Guendouz et al. 2021; Wang et al. 2022; Yuan et al. 2022; Taha et al. 2024), whereas we have directly measured the fluorescence of acetone‐extracted Chl. Although SPAD‐502 readings and extraction‐based Chl quantification methods correlate strongly, with *R* ^*2*^ values larger than 0.9 (Markwell et al. 1995; Castelli et al. 1996), the two methods still show slight differences, which could partially explain why some CVIs perform better in our study compared to previous research and vice versa. Despite these differences, our data shows that several visual indexes accurately quantify Chl content, and that the Rn, G:R, and NDI indexes are all reliable, with slight differences in the top‐performing CVI for each species.

### 3.2 GWAS Using GreanLeafVI Accurately Identifies Chlorophyll Biosynthesis Genes

To test whether visual indexes are sufficiently accurate to identify phenotypic differences between genotypes, we ran a GWAS on leaf Chl content in lettuce, the plant species where the use of digital images correlated most strongly with mg Chl cm^−2^. We used a panel of 184 lettuce cultivars grown in controlled conditions and harvested the fourth leaf 10 days after emergence for three plants of each genotype. We then imaged a 30 mm leaf disk of this leaf, ran the GreenLeafVI plugin on the images thus generated, and used the results for subsequent analyses. We used an extensive SNP dataset to run GWASs on G:R (the best‐performing CVI for lettuce) and Rn (the overall best‐performing CVI) as a measure of Chl content. From these GWASs, a significant peak on chromosome 4 was identified (Figure 2E, Figures S9 and 10), which corresponded to the peak found by Zhang, Qian, et al. (2022) in their GWAS on 125 lettuce genotypes using Chl measurements on leaf extracts. This peak was shown to be associated with the lettuce *Golden‐Like (LsGLK)* gene, which is an important regulator of chloroplast development (Zhang, Qian, et al. 2022). The association of the significant peak that was observed in all three GWASs with a gene regulating chloroplast development explains the variation in Chl content that was observed by either direct Chl measurement (Zhang, Qian, et al. 2022) or, in our case, by using digital images as a reference. The similarity in outcomes between the two GWASs clearly shows that our G:R and Rn data can reproduce the association between the Chl content phenotype and the SNP located near the *LsGLK* gene, indicating that these CVIs accurately quantify Chl content and offer a reliable method to identify a phenotypic vs. genotypic association.

## 4 Concluding Remarks

Our results show that the GreenLeafVI plugin provides an easy‐to‐use and reliable tool for high‐throughput digital image‐based quantification of Chl content in leaves of different plant species. Importantly, image‐based Chl quantification is non‐destructive, allowing researchers to monitor the same plant over time, and digital images can be easily stored and re‐examined later. Other traits besides Chl content (e.g., anthocyanin production, leaf shape and size, etc.) can be measured using digital images, and such traits may be examined at any moment, whereas this information is lost when using destructive methods of Chl quantification. Thus, image‐based Chl quantification offers additional benefits compared to other methods. Taking digital images and automated image processing are also less labor‐intensive, less costly, and a more sustainable alternative to large‐scale Chl extractions. Considering the various benefits of digital image‐based Chl quantification, we propose that this method be used in future applications.

## Author Contributions

T.L., J.v.L., and R.O. designed the experiments. T.L. and J.v.L. performed Chl extraction experiments, and J.v.L. performed lettuce phenotyping for GWAS. T.L. performed the correlation analyses, and S.L.M. and B.L.S. performed GWAS analysis. J.v.L., T.L., and J.W. wrote the ImageJ macro and Java scripts. T.L. wrote the manuscript with input from all other authors.

## Supporting information


**Figure S1:** Correlation between Green: Red ratio and Chl content in Arabidopsis, tomato, tobacco, and lettuce. Correlation analysis was done by linear regression using a model where *y* = *x*.
**Figure S2:** Correlation between the Kawashima Index and Chl content in Arabidopsis, tomato, tobacco, and lettuce. Correlation analysis was done by linear regression using a model where *y* = *x*.
**Figure S3:** Correlation between the Normalized Red value (Rn) and Chl content in Arabidopsis, tomato, tobacco, and lettuce. Correlation analysis was done by linear regression using a model where *y* = *x*.
**Figure S4:** Correlation between the Normalized Green value (Gn) and Chl content in Arabidopsis, tomato, tobacco, and lettuce. Correlation analysis was done by linear regression using a model where *y* = *x*.
**Figure S5:** Correlation between Normalized Blue value (Rn) and Chl content in Arabidopsis, tomato, tobacco, and lettuce. Correlation analysis was done by linear regression using a model where *y* = *x*.
**Figure S6:** Correlation between the Normalized Difference Index (NDI) and Chl content in Arabidopsis, tomato, tobacco, and lettuce. Correlation analysis was done by linear regression using a model where *y* = *x*.
**Figure S7:** Correlation between the Green Leaf Index (GLI) and Chl content in Arabidopsis, tomato, tobacco, and lettuce. Correlation analysis was done by linear regression using a model where *y* = *x*.
**Figure S8:** Correlation between the Woebbecke Index and Chl content in Arabidopsis, tomato, tobacco, and lettuce. Correlation analysis was done by linear regression using a model where $y=-\frac{1}{x}$.
**Figure S9:** Quantile‐Quantile plot for Green: Red ratio on the fourth leaf of 184 lettuce cultivars.
**Figure S10:** Manhattan plot of the GWAS on the Normalized Red value measured on the fourth leaf of 184 lettuce cultivars. Genomic position, indicated in megabasepairs (Mbp), is shown on the *x*‐axis, chromosome numbers are indicated on top. Significance as –log_10_(*p*) is shown on the y‐axis. The Bonferroni threshold of –log_10_(*p*) > 7.69 is indicated by the red horizontal line.
**Protocol S1.** User guide for GreenLeafVI plugin.

## Data Availability

The data that support the findings of this study are available from the corresponding author upon reasonable request. The GreenLeafVI source code, documentation, and further information are available at https://github.com/jelmervanlieshout/GreenLeafVI.
