# Supplementary material for: GreenLeafVI: A FIJI Plugin for High‐Throughput Analysis of Leaf Chlorophyll Content
Source: Physiol Plant. 2025 Oct 19;177(5):e70588. doi: 10.1111/ppl.70588 (PMC12535850; doi:10.1111/ppl.70588)
Supplement: Supplementary file 1 — Figure S1: Correlation between Green: Red ratio and Chl content in Arabidopsis, tomato, tobacco, and lettuce. Correlation analysis was done by linear regression using a model where y = x. Figure S2: Correlation between the Kawashima Index and Chl content in Arabidopsis, tomato, tobacco, and lettuce. Correlation analysis was done by linear regression using a model where y = x. Figure S3: Correlation between the Normalized Red value (Rn) and Chl content in Arabidopsis, tomato, tobacco, and lettuce. Correlation analysis was done by linear regression using a model where y = x. Figure S4: Correlation between the Normalized Green value (Gn) and Chl content in Arabidopsis, tomato, tobacco, and lettuce. Correlation analysis was done by linear regression using a model where y = x. Figure S5: Correlation between Normalized Blue value (Rn) and Chl content in Arabidopsis, tomato, tobacco, and lettuce. Correlation analysis was done by linear regression using a model where y = x. Figure S6: Correlation between the Normalized Difference Index (NDI) and Chl content in Arabidopsis, tomato, tobacco, and lettuce. Correlation analysis was done by linear regression using a model where y = x. Figure S7: Correlation between the Green Leaf Index (GLI) and Chl content in Arabidopsis, tomato, tobacco, and lettuce. Correlation analysis was done by linear regression using a model where y = x. Figure S8: Correlation between the Woebbecke Index and Chl content in Arabidopsis, tomato, tobacco, and lettuce. Correlation analysis was done by linear regression using a model where y=−1x. Figure S9: Quantile‐Quantile plot for Green: Red ratio on the fourth leaf of 184 lettuce cultivars. Figure S10: Manhattan plot of the GWAS on the Normalized Red value measured on the fourth leaf of 184 lettuce cultivars. Genomic position, indicated in megabasepairs (Mbp), is shown on the x‐axis, chromosome numbers are indicated on top. Significance as –log10(p) is shown on the y‐axis. The Bonferroni threshold of –log1 [file PPL-177-e70588-s001.pdf]

GreenLeafVI: A FIJI plugin for high-throughput analysis of leaf chlorophyll content

Thalia Luden, Jelmer van Lieshout, Sarah L. Mehrem, Basten L. Snoek, Joost Willemse, Remko Offringa

Supplementary Data

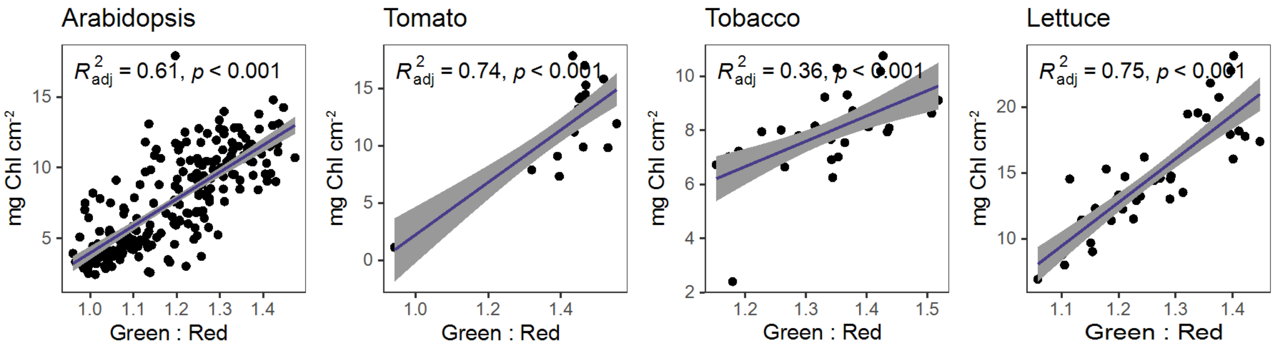

Figure S1: Correlation between Green : Red ratio and Chl content in Arabidopsis, tomato, tobacco, and lettuce. Correlation analysis was done by linear regression using a model where $y = x$.

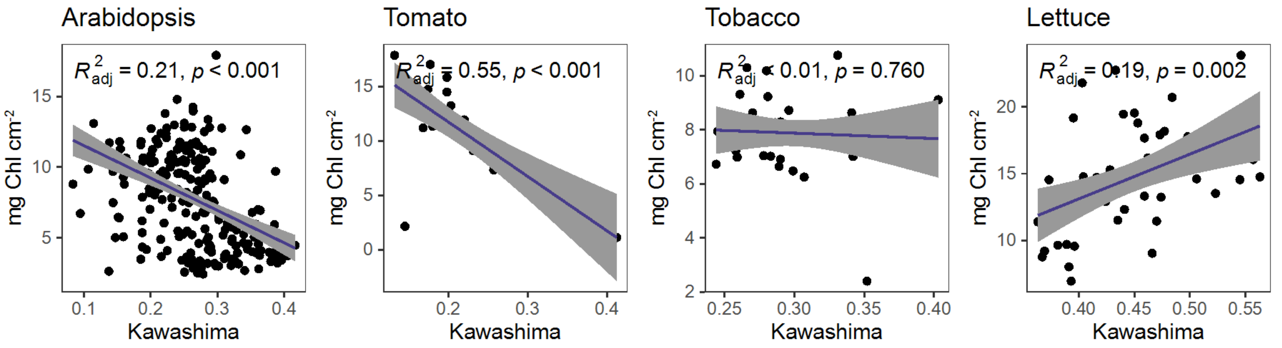

Figure S2: Correlation between the Kawashima Index and Chl content in Arabidopsis, tomato, tobacco, and lettuce. Correlation analysis was done by linear regression using a model where $y = x$.

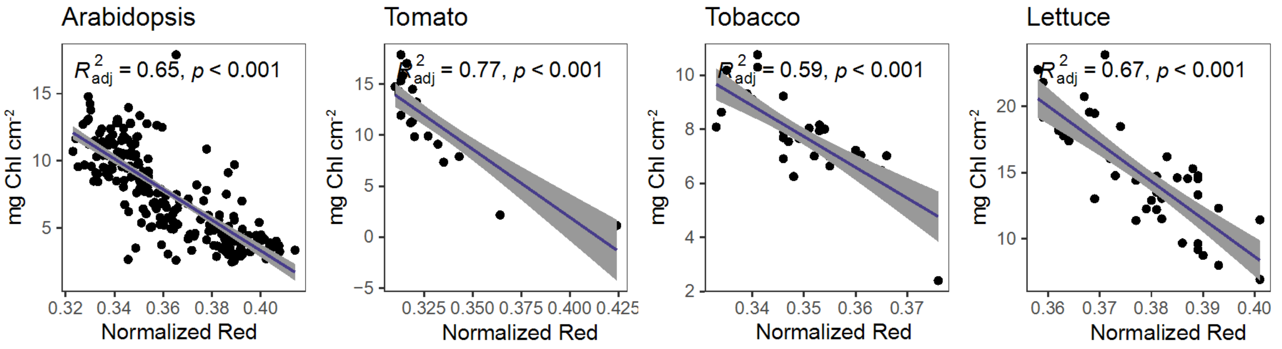

Figure S3: Correlation between the Normalized Red value (Rn) and Chl content in Arabidopsis, tomato, tobacco, and lettuce. Correlation analysis was done by linear regression using a model where $y = x$.

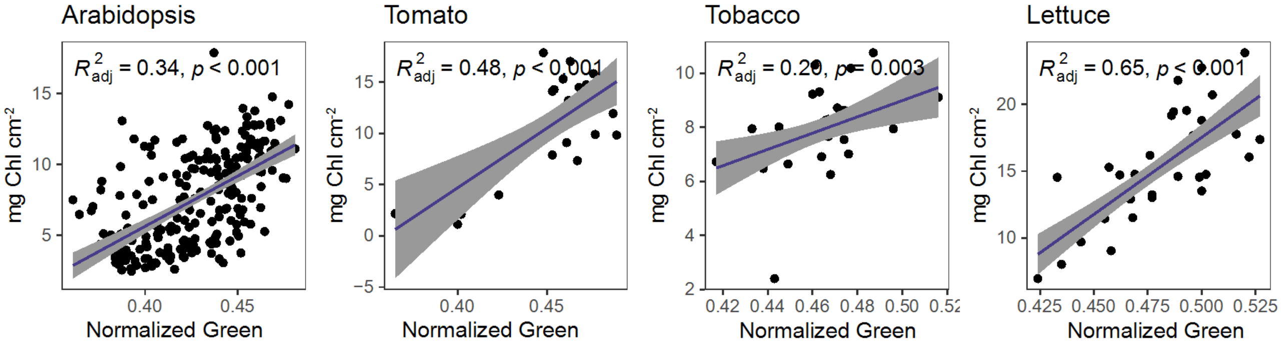

Figure S4: Correlation between the Normalized Green value (Gn) and Chl content in Arabidopsis, tomato, tobacco, and lettuce. Correlation analysis was done by linear regression using a model where $y = x$.

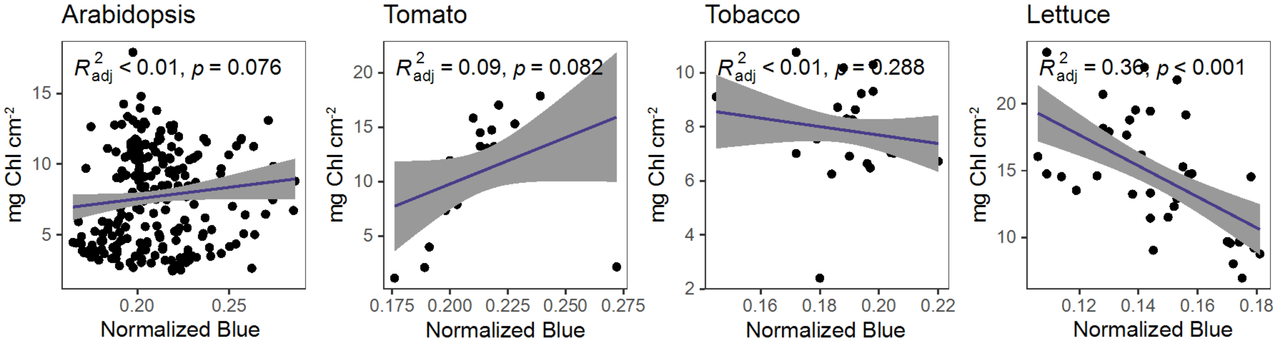

Figure S5: Correlation between Normalized Blue value (Rn) and Chl content in Arabidopsis, tomato, tobacco, and lettuce. Correlation analysis was done by linear regression using a model where $y = x$.

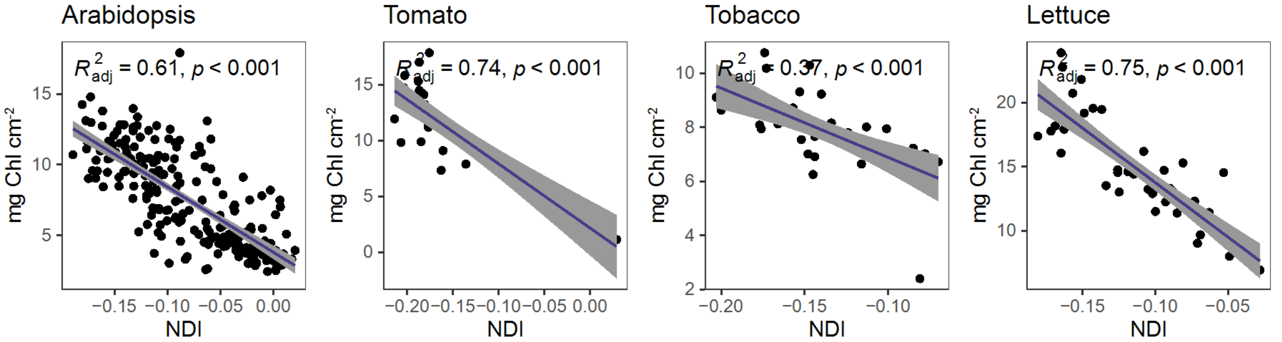

Figure S6: Correlation between the Normalized Difference Index (NDI) and Chl content in Arabidopsis, tomato, tobacco, and lettuce. Correlation analysis was done by linear regression using a model where $y = x$.

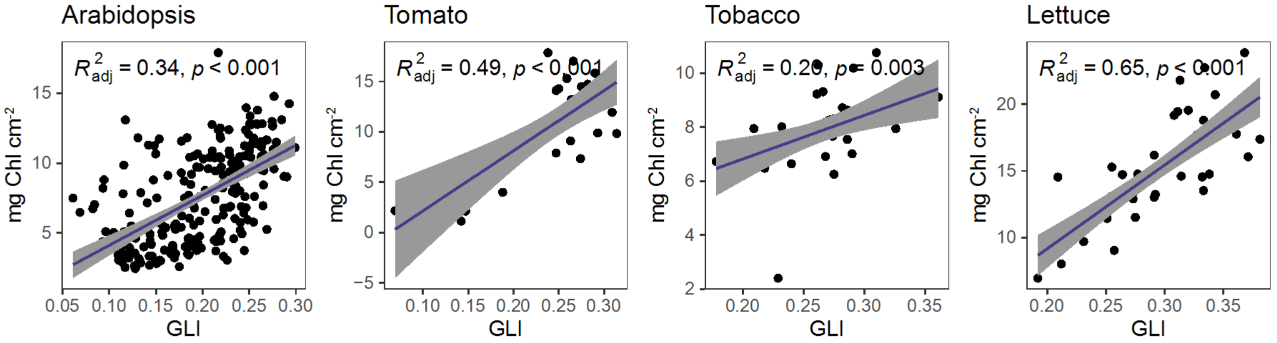

Figure S7: Correlation between the Green Leaf Index (GLI) and Chl content in Arabidopsis, tomato, tobacco, and lettuce. Correlation analysis was done by linear regression using a model where $y = x$.

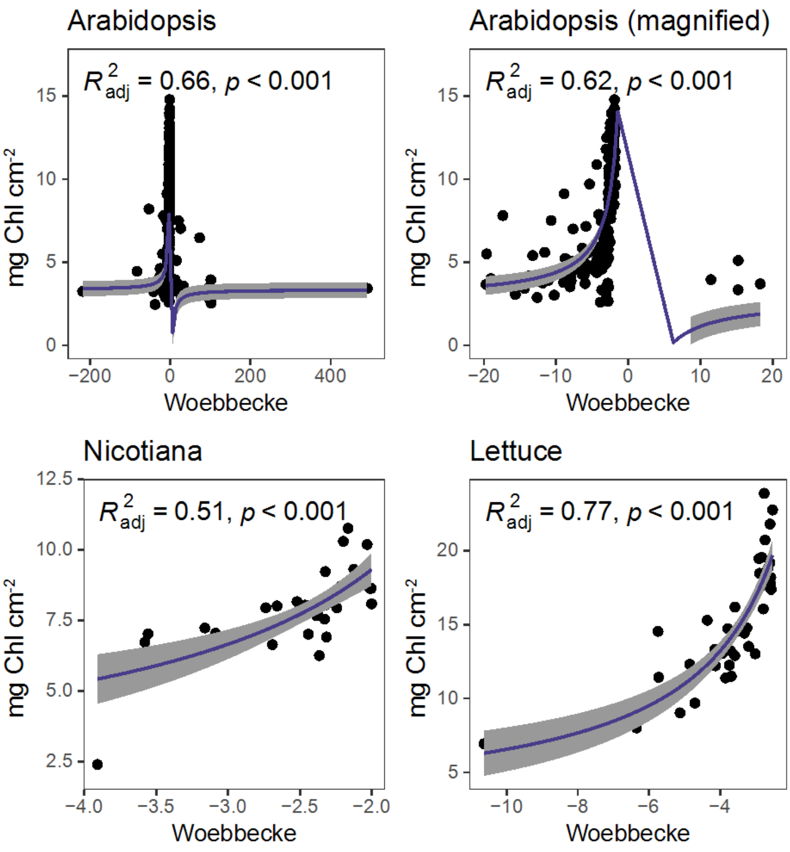

Figure S8: Correlation between the Woebbecke Index and Chl content in Arabidopsis, tomato, tobacco, and lettuce. Correlation analysis was done by linear regression using a model where $y = -\frac{1}{x}$.

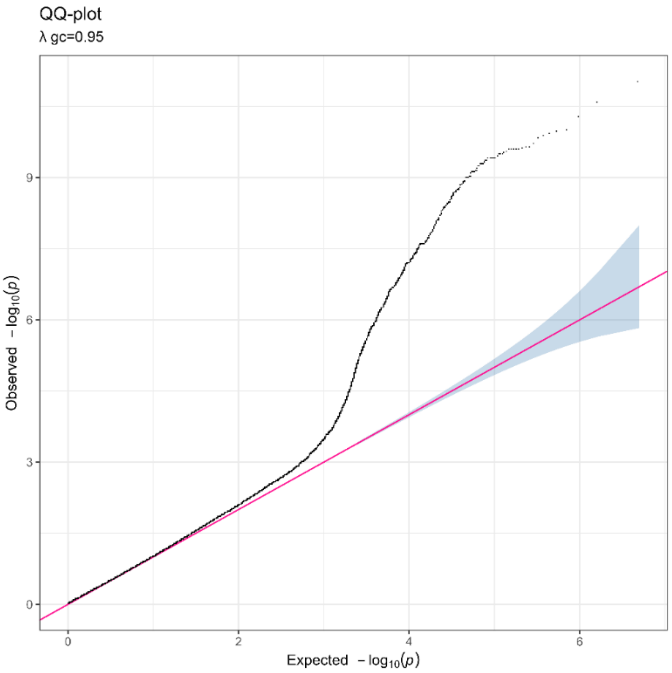

Figure S9: Quantile-Quantile plot for Green : Red ratio on the fourth leaf of 184 lettuce cultivars.

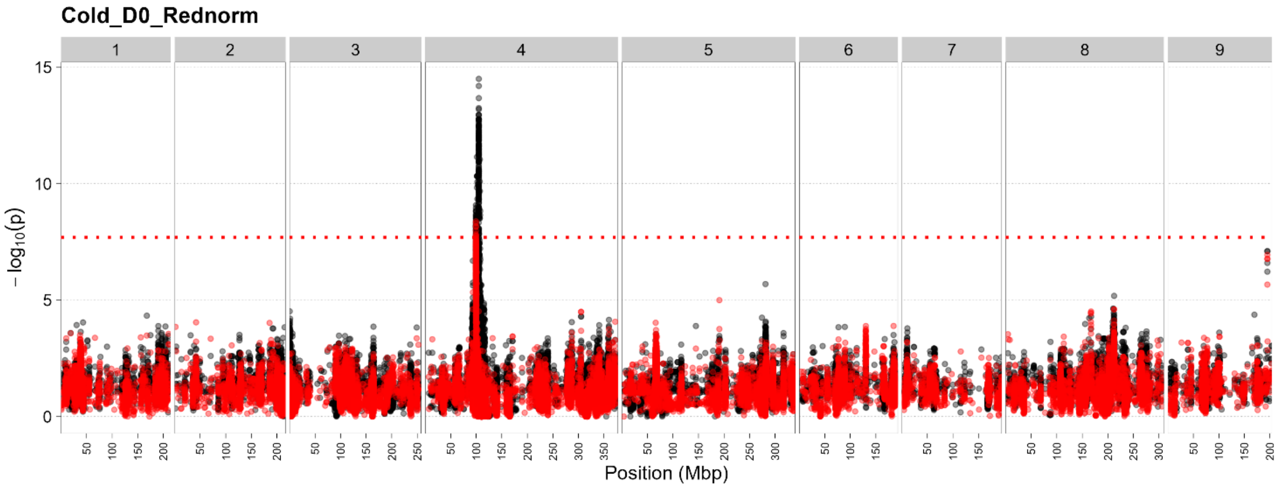

Figure S10: Manhattan plot of the GWAS on the Normalized Red value measured on the fourth leaf of 184 lettuce cultivars. Genomic position, indicated in megabasepairs (Mbp), is shown on the x-axis, chromosome numbers are indicated on top. Significance as $-\log_{10}(p)$ is shown on the y-axis. The Bonferroni threshold of $-\log_{10}(p) > 7.69$ is indicated by the red horizontal line.

Protocol S1: GreenLeafVI user guide

Imaging setup and white balancing

The main limitation to image-based measurement is the variation in brightness and contrast between images. In order to calibrate the image brightness to reduce inter-image variation, we included an optional white-balancing step in the GreenLeafVI plugin (figure 1). The white-balancing step requires that a white reference area be photographed under the same conditions as the images to be analysed. This ensures that all images are white balanced to the same reference, reducing the variation in brightness between images that can arise from differential lighting or exposure settings during the imaging process. For this purpose, we recommend including a small white reference square or circle of a known size (e.g. 4 cm^2) in each of the images. This will serve as a reference for both the white balancing and for setting the image scale (via Analyze > set scale), which is useful for measuring the leaf area in addition to its Chl content. Alternatively, a single white reference area could be photographed at the start or end of the series of images to calibrate the entire set, assuming that lighting and exposure conditions are constant throughout the process.

When running the white balancing step, the user can select an input- and output folder and can select whether the images should be calibrated individually or batch-calibrated based on a single white reference image. Individual white balancing requires the user to select the reference area in each of the images and is therefore more time-consuming than the batch calibration, but is also more accurate and suitable for comparing images where the lighting, exposure, and focal distances are not constant. After the user has chosen whether to process images individually or in batch, the image is split into Red, Green, and Blue channels and the mean pixel intensity in the each channel is automatically calculated in the selected white area. Next, the pixel adjustment factor for each of the channels is calculated by dividing the maximum brightness of 8-bit images (255) by the mean pixel intensity of the white area (adj_factor = 255/mean). The white reference is then set to an intensity of 255 in all three channels, and the pixels outside of the reference area are then normalized based on the adjustment factor. Finally, the image is re-stacked to an 8-bit RGB image and saved in a folder selected by the user, and the image is saved under its original name with addition of a "_whitebalanced" suffix.

For optimal reproducibility between images, we recommend imaging under constant lighting with a uniform background that shows sufficient contrast to the leaves that will be imaged such as a black, white, light blue, or orange background. In addition, variation between images can be further reduced by using the same focal distance between the camera and the leaves or plants that will be imaged throughout the process. If multiple objects are to be measured in one image, it is important that these do not overlap if a separate measurement of each object is desired.

Segmentation of images to reduce background

In order to exclude measurements of non-leaf objects or areas in the background of the image, we included an optional segmentation step in the GreenLeafVI plugin (figure 1). This process uses the difference in colour between the leaves and the background to extract only leaf-like objects. Because of this, the segmentation performs better when a background with an even colour that has sufficient contrast to the leaves is used during imaging, such as black, white, light blue, or orange.

In order to run the segmentation process, the user needs to select an input- and output folder, and can adjust the settings for minimum object area, as well as minimum- and maximum values in the hue saturation value colour space (HSV). While the settings included in our plugin should perform well for any leaf-like object, we recommend that users customize these settings to the object of their interest to optimise segmentation performance. When the segmentation step is performed, a mask is created based on the HSV and minimum area inputs, and used to remove the background which is set to 0. The images are then saved in a folder selected by the user under their original name extended by a "_segmented" suffix.

RGB analysis and colour-based methods of chlorophyll estimation

The main feature of the GreenLeafVI plugin is the measurement of pixel intensity of individual objects (leaves) in the Red, Green, and Blue channels and the calculation of various colorimetric visual indexes (CVIs) from these data (figure 1C). The RedGreenBlue measurement can be run on all images saved in a single folder at once (we analyzed up to 446 images at a time), and the results are saved as a .csv file in the same folder. Images that can be used as input are preferably white-balanced and segmented, but the measurements can be run on unprocessed images as well, although this may result in poor identification of individual objects.

To measure the R, G, and B pixel intensities of individual objects in an image, a mask is made that selects individual objects in a similar manner as in the segmentation step. It then splits the original image into Red, Green, and Blue channels and overlays the mask on each of the channels. The minimum, maximum, median, and mean pixel intensity of each object of the mask is then measured for each of the channels, and written to the results file. The mean values of the Red, Green, and Blue channels are used to calculate the different visual indexes that are used to estimate Chl content: Green:Red ratio (GR_ratio), Red:Green ratio (RG_ratio), Kawashima index (Kawashima), Green Leaf Index (GLI), Normalized Difference Index (NDI), normalized Red (Red_norm), normalized Green (Green_norm), normalized Blue (Blue_norm), and the Woebbecke index (Woebbecke). The results file also contains a column with the image name, the region of interest (ROI), which refers to individual objects in the image, their area, and an x- and y- coordinate that can be used to retrace to which object a measurement refers.

Since ImageJ scans for objects in a left-to-right and top-to-bottom order, the top left object will be the first to be detected and the bottom-right the last. This can be taken into account when preparing multiple leaves in one image, for

example by making sure that the objects are ordered top-to-bottom or by arranging the objects in a diagonal from left to right (figure 1). Alternatively, each leaf or plant can be imaged individually. For high-throughput purposes, we advise creating a metadata file that links the image name to the content of the image, which can then be merged with the results file for further analysis.
